# DNA-Mediated Stack Formation of Nanodiscs

**DOI:** 10.3390/molecules26061647

**Published:** 2021-03-16

**Authors:** Madhumalar Subramanian, Charlotte Kielar, Satoru Tsushima, Karim Fahmy, Jana Oertel

**Affiliations:** 1 Biophysics Department, Institute of Resource Ecology, Helmholtz-Zentrum Dresden-Rossendorf, Bautzner Landstrasse 400, 01328 Dresden, Germany; m.subramanian@hzdr.de (M.S.); c.kielar@hzdr.de (C.K.); s.tsushima@hzdr.de (S.T.); 2 Center for Molecular and Cellular Bioengineering, Technische Universität Dresden, 01062 Dresden, Germany

**Keywords:** membrane-scaffolding protein, nanodisc, membrane protein, lipid bilayer, lipid protein interaction, multimerization, self-assembly, bionanotechnology

## Abstract

Membrane-scaffolding proteins (MSPs) derived from apolipoprotein A-1 have become a versatile tool in generating nano-sized discoidal membrane mimetics (nanodiscs) for membrane protein research. Recent efforts have aimed at exploiting their controlled lipid protein ratio and size distribution to arrange membrane proteins in regular supramolecular structures for diffraction studies. Thereby, direct membrane protein crystallization, which has remained the limiting factor in structure determination of membrane proteins, would be circumvented. We describe here the formation of multimers of membrane-scaffolding protein MSP1D1-bounded nanodiscs using the thiol reactivity of engineered cysteines. The mutated positions N42 and K163 in MSP1D1 were chosen to support chemical modification as evidenced by fluorescent labeling with pyrene. Minimal interference with the nanodisc formation and structure was demonstrated by circular dichroism spectroscopy, differential light scattering and size exclusion chromatography. The direct disulphide bond formation of nanodiscs formed by the MSP1D1_N42C variant led to dimers and trimers with low yield. In contrast, transmission electron microscopy revealed that the attachment of oligonucleotides to the engineered cysteines of MSP1D1 allowed the growth of submicron-sized tracts of stacked nanodiscs through the hybridization of nanodisc populations carrying complementary strands and a flexible spacer.

## 1. Introduction

The design of nanometer-sized discoidal lipid bilayers has increasingly gained attention over the last decade. Membrane-scaffolding proteins (MSPs) [1], amphipathic polymers [2,3,4] and alkylated circular dsDNA [5] have been shown to be capable of stabilizing nano-sized discoidal lipid bilayers. Such systems can harbor single membrane proteins of defined oligomeric states of membrane proteins in a native lipid environment. The high protein to lipid ratio as compared to vesicles and the lack of inside out ambiguity of membrane protein orientation is advantageous in a variety of functional studies, which can be performed at the single molecule level in nano-sized lipid bilayers, as shown [6]. In principle, the structure determination of membrane proteins by diffraction-based experiments could also be achieved with single molecules. The continuing size reduction of membrane protein crystals for X-ray analyses demonstrates impressively the technical development toward the physical limit of X-ray diffraction [7,8]. Designed nano-sized lipid bilayers ultimately appear particularly suited for efforts toward single membrane protein diffraction. Such self-assembled systems preserve in a single particle not only highly specific lipid protein interactions, but also those that originate in functionally important global properties of lipid bilayers, such as thickness, fluidity, curvature strain, lateral pressure and membrane protein hydration [9]. These types of interactions are often absent or replaced by non-bilayer-like lipid protein interactions in membrane protein crystals. Although the independence of protein crystallization is the main driving force for research into the design of novel lipid bilayer nanoparticles, the increase in scattering intensity with the square of the particle number also motivates the design of either crystalline or multimeric assemblies of such lipid–protein particles. Given the evident value of their potential use in crystallization-independent membrane protein structural biology, the study of differently stabilized nano-sized lipid bilayers themselves has become a field of research in many respects, e.g., addressing stability, lipid phase transitions and lipid exchange dynamics [10,11,12,13,14,15,16].

Nanodiscs are composed of nano-sized phospholipid bilayers encircled by two copies of an α-helical amphipathic MSP [17,18,19]. MSPs are truncated forms of the human apolipoprotein A-I (ApoA-I) [20], which is the major protein component of high-density lipoprotein (HDL) particles [21]. Whereas the MSP1D1 variant has become a widely used system and reference for other nano-sized lipid bilayer particles, longer apolipoproteins have been used recently for the crystallization of empty lipid bilayers [22]. We surmise that ultimately, the rationally designed chemical linkage of nanodiscs can be combined with crystallization to generate larger geometrically defined assemblies of membrane proteins in their native lipid environment. Such systems would enhance scattering amplitudes in diffraction studies and bridge the gap between single particles and conventional crystals. The inherent advantage applies to both X-ray analyses as well as to the more recent cryo-electron microscopy (cryo-EM) method using microcrystal electron diffraction [23].

Here, we describe experiments that aim at producing multimeric one-dimensional assemblies of nanodiscs in a crystallization-independent fashion. For this purpose, cysteines were introduced into MSP1D1. While human ApoA-I is free of cysteines, other artificial cysteine mutations of ApoA-I have been characterized [24], among which N74C and K195C were found to lie close to opposite to each other across the circumference of the nanodisc scaffold. We substituted cysteines for the homologous Asn-42 and Lys-163 residues in MSP1D1, resulting in the variants MSP1D1_N42C, MSP1D1_K163C and the double mutant MSP1D1_N42C/K163C. The variants enabled the direct disulphide bond formation between nanodiscs as well as DNA-hybridization between nanodiscs with oligonucleotides attached to their cysteine thiols. Both approaches supported nanodisc multimer formation with remarkably different efficiencies and geometrical arrangements. The DNA-mediated linkage appeared particularly attractive for the formation of submicron-sized supramolecular assemblies of nanodiscs.

## 2. Results

### 2.1. Expression and Purification of MSP1D1 Cysteine Variants

**Figure 1:**
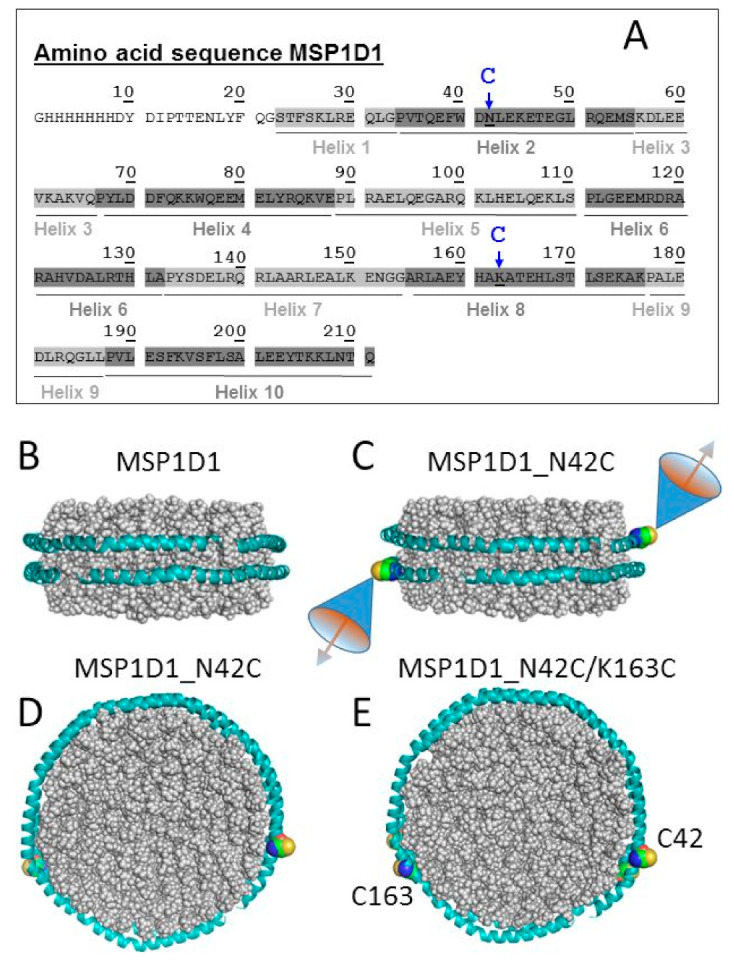
Amino acid sequences of membrane-scaffolding protein (MSP)1D1 variants and their putative structures of nanodiscs. (**A**) Cysteine substitutions at positions N42 and K163 are indicated by arrows. The α-helical segments of MSP1D1 (Helix 1 to Helix 10) are highlighted. In all experiments, the MSP1D1 variants kept their N-terminal His-tags used for affinity purification. (**B**) Hypothetical model of the MSP1D1 nanodisc generated with Nanodisc Builder [25,26]. (**C**) Model of the nanodisc formed by the MSP1D1_N42C variant, mutated cysteine (Cys) residues included with Pymol [27]. The cones exemplify the geometrical relations between an arbitrarily chosen steric angle of orientations of a thiol-linked chemical modification in the two MSP1D1_N42C monomers. The model emphasizes the antiparallel direction of the molecular axes of a chemical modification at the two cysteines with respect to the bilayer normal. (**D**) The previous model in top view. (**E**) Top view of the nanodisc model of the MSP1D1_N42C/K163C variant showing the close proximity of the pairs of cysteines (C42 from one and C163 from the other monomer). The spheres of the space-filling representations of the cysteines have been enlarged for better visibility.

To enable the assembly of individual nanodiscs into multimeric structures, we introduced cysteines into MSP1D1 to induce direct site-specific disulfide bond formation or exploit their thiol reactivity for the attachment of maleimide-conjugated oligonucleotides. We have introduced the amino acid replacements N42C and K195C into the MSP1D1 sequence (Figure 1A). Based on the proposed molecular model of the MSP1D1 nanodisc [25,26] (Figure 1B), the introduced cysteines would lie roughly in opposition to each other (Figure 1C–E). MSP1D1, the two single amino acid replacement variants MSP1D1_N42C, MSP1D1_K163C and the doubly mutated variant MSP1D1_N42C/K163C were purified and analyzed by sodium dodecyl sulphate-polyacrylamide gel electrophoresis (SDS-PAGE) (Figure S1). The two single cysteine variants generated bands at the monomer molecular weight of 25 kDa, whereas the double mutation N42C/K163C exhibited an additional fraction of dimers at 50 kDa even at reducing conditions (1 mM of tris(2-carboxyethyl)phosphine (TCEP)). Under non-reducing conditions, all mutated cysteine variants migrated as coexisting dimeric and monomeric forms. The nanodisc assembly of the MSP1D1 variants was performed under reducing conditions (Materials and Methods). The elution profile obtained with size exclusion chromatography (SEC) showed that all MSP1D1 cysteine variants self-assembled with lipids into particles covering the expected size range of MSP1D1 nanodiscs (Figure S2).

### 2.2. Secondary Structure and Particle Size of Nanodiscs

**Figure 2:**
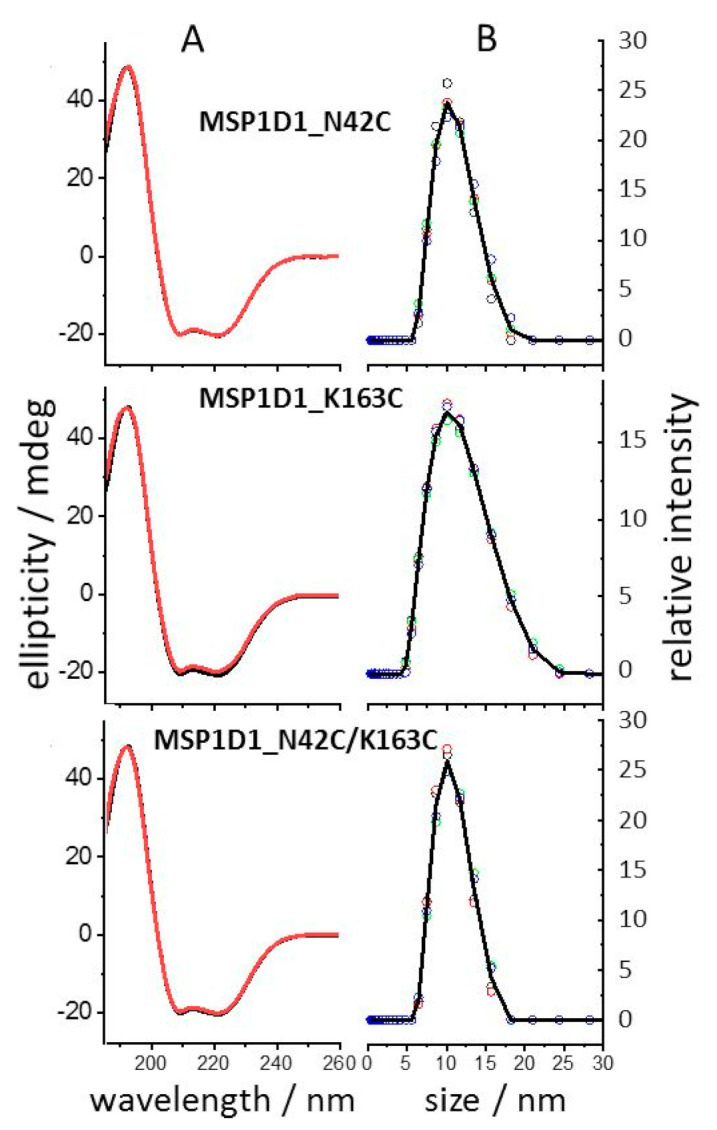
Secondary structure of MSP1D1variants and hydrodynamic radii of their corresponding nanodiscs. (**A**) Circular dichroism (CD) spectra of 1, 2-dimyristoyl-*sn*-glycero-3-phospho-(1′-rac-glycerol) (DMPG)-reconstituted nanodiscs from different MSP1D1 variants (5 µM in buffer A). The spectral features of the mutated MSP1D1 variants (red) were superimposable with the CD spectrum of MSP1D1 (black) evidencing the absence of measurable secondary structural aberrations in the cysteine-containing variants. Averages of independently measured spectra (*n* = 8) are shown. (**B**) Dynamic light scattering (DLS) analysis revealed the expected hydrodynamic radius of ~10 nm diameter found for MSP1D1. Four iterative measurements were performed at 25 °C (open circles) and averaged (solid lines).

The secondary structures of the MSP1D1 variants in the assembled 1, 2-dimyristoyl-*sn*-glycero-3-phospho-(1′-rac-glycerol) (DMPG)-filled nanodiscs were compared by circular dichroism (CD). Figure 2A shows that the spectra of all nanodisc preparations were superimposable with the spectrum of the MSP1D1 nanodisc measured under identical conditions and confirmed the high content of α-helical structure evident from the large 220 nm to 208 nm peak ratio in all spectra. The nanodisc size was assessed by dynamic light scattering (DLS) under reducing conditions (1 mM of TCEP) and compared with that of conventional MSP1D1 nanodiscs (Figure 2B). The average hydrodynamic diameter (DH) derived from this was close to 10 nm, i.e., in good agreement the reported values, e.g., [20,28]. These results show that neither the protein structure nor the self-assembly process of the nanodisc formation were affected by the cysteine mutations in any of the MSP1D1 variants.

### 2.3. Thiol Reactivity of Cysteines in Engineered Nanodisc Variants

**Figure 3:**
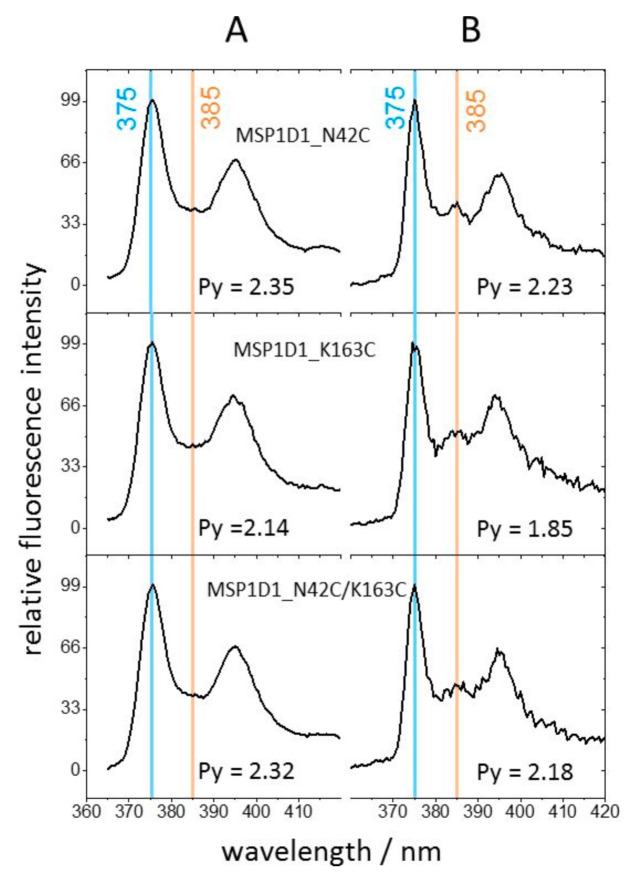
Fluorescence emission spectra of pyrene-labeled nanodiscs. The thiol group of the cysteines in the three MSP1D1 variants was labelled with the solvatochromic fluorophore N-1-pyrene maleimide and excited at 345 nm. The intensities of the characteristic emission peaks at 375 nm and 385 nm define the value Py = I_375_/I_385_ which reports the degree of aqueous exposure of the fluorophore (Py > 1 indicating a hydrophilic environment). (**A**) Nanodiscs were directly labeled with pyrene or (**B**) pre-labeled MSP1D1 variants were used for nanodisc assembly.

To address the accessibility of the engineered cysteines for biochemical modification, we used fluorescent labelling with N-1-pyrene maleimide. Pyrene emission arises from its π* -> π transition, generating five major emission bands between ~375 and 410 nm [29,30]. The relative intensity of the 375 nm and 385 nm emissions corresponds to the first (0–0) and third (0–2) vibronic transition, respectively. The intensity ratio Py = I_375_/I_385_ is widely used for determining protein changes in microenvironments by reacting N-1-pyrene maleimide with cysteine thiols [30,31]. Particularly, pyrene has been shown to be an excellent environmental dipole sensor and is relatively insensitive to hydrogen bonding [32,33]. Thus, a value of Py < 1 represents a hydrophobic environment, whereas Py > 1 corresponds to a hydrophilic environment [34]. Figure 3 shows the emission spectra of nanodiscs generated with the three MSP1D1 variants by two labelling methods. The cysteines were reacted with N-1-pyrene maleimide either after (Figure 3A) or before nanodisc formation (Figure 3B). In both cases, the 375 nm emission was the most prominent band, whereas emission at 385 nm was generally much weaker, indicating a predominant hydrophilic exposure of the fluorophore, irrespective of the labelling method. In the case of labelling the MSP1D1 variants before nanodisc formation (Figure 3B), however, a well-resolved small emission peak at 385 nm was reproducibly observed. This is a strong indication that the introduced cysteine side chains in all MSP1D1 variants exhibited sufficient flexibility to let the pyrene moiety partially sense the lipophilic environment upon the association of the pre-labelled scaffold protein with the lipid bilayer. In contrast, the fluorescent labelling of previously assembled nanodiscs did not give rise to a well resolved 385 nm emission peak. Thus, labelling appeared to be restricted to side chain orientations that supported the more prevalent aqueous exposure of the thiols, whereas thiols closer to the hydrophobic core of the nanodiscs were inaccessible for fluorescent labelling. Clearly, the majority of cysteines at both mutated positions supported the exposure of the reactive thiols to the outer hydrophilic circumference of the protein scaffold. Thus, the formation of covalently linked nanodisc multimers through thiol chemistry appeared possible. However, the data also demonstrate the coexistence of at least two geometries of the proteins with respect to the encircled lipid bilayer in all of the cysteine-carrying MSP1D1 variants. Furthermore, the absence of the characteristic excimer band of pyrene above 450 nm [35] in all fluorescence spectra (not shown) precludes that any two cysteines in the assembled scaffolds formed long-lived neighbors (distance < 1 nm) [30].

### 2.4. Nanodisc Multimers of MSP1D1 Variants

**Figure 4:**
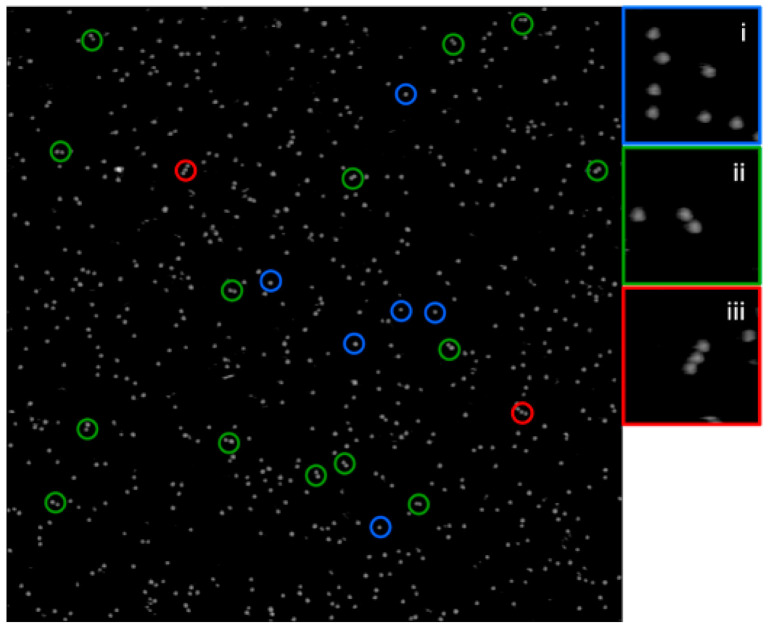
Atomic force microscopy (AFM) analysis of the disulphide-linked oligomer formation of nanodiscs. The full-sized AFM image (2 × 2 µm^2^ at a height scale of 7.5 nm) shows an example of manual nanodisc counting of liquid mode AFM images. Monomers, dimers and trimers of nanodiscs are encircled in blue, green and red, respectively, and shown enlarged to the right. Data are from the MSP1D1_N42C variant. A full statistical analysis for all variants is given in Supplemental Materials, Figure S4 and Tables S3–S6.

**Figure 5:**
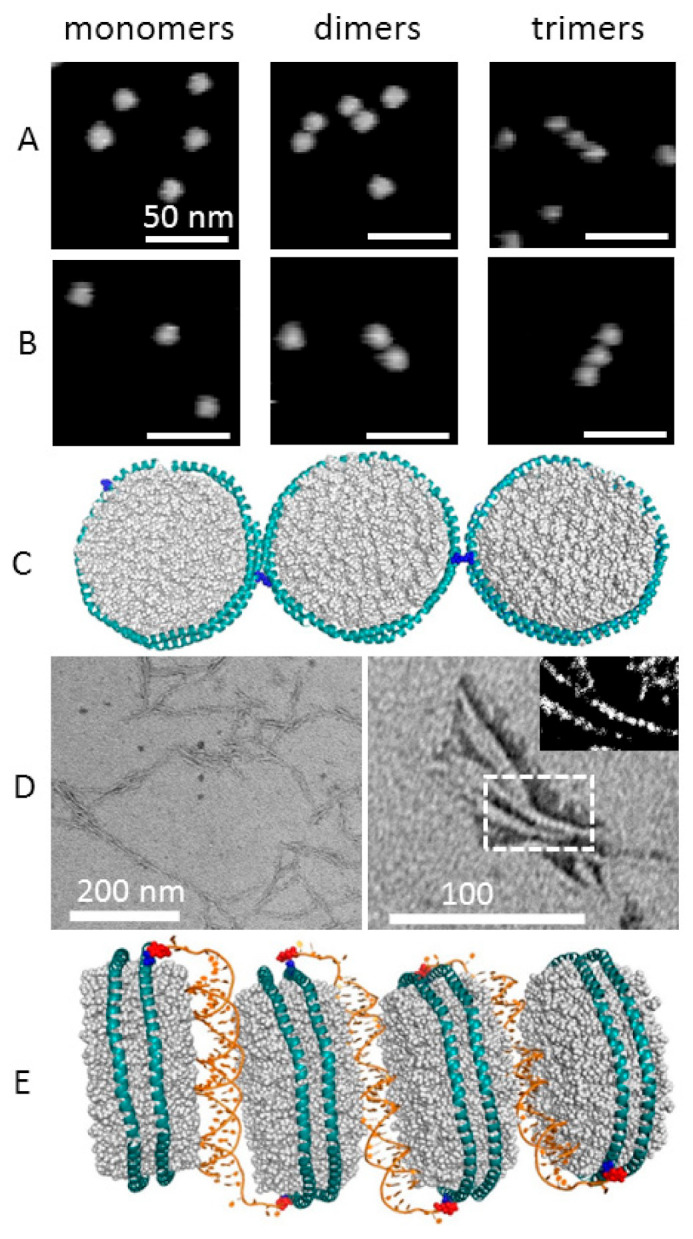
Imaging of nanodisc multimers. (**A**) AFM images of disulphide-bond linked nanodisc multimers formed by copper-catalyzed disulphide bond formation between individual MSP1D1_N42C nanodiscs. (**B**) The corresponding multimers formed by MSP1D1_N42C/K163C nanodiscs. (**C**) Molecular model of disulphide-linked nanodiscs. (**D**) Left panel: representative TEM image of > 200 nm long threads of DNA-linked MSP1D1_N42C nanodiscs. Right panel: close-up TEM image of a short multimeric assembly of the same nanodisc preparation. The inset shows a contrast-enhanced version (using gray scale threshold Huang representation in ImageJ) of the boxed image area. (**E**) Putative molecular model of the multimeric nanodisc assembly. Cys-mutated MSP1D1 and maleimide-modified oligos were alternately arranged to constitute a multimeric structure in accordance with the TEM images (see subsection structure modeling for details).

The variants MSP1D1_N42C and MSP1D1_N42C/K163C were subjected to copper-catalyzed disulphide bond formation between the solvent-accessible cysteines of multiple nanodiscs of a given MSP1D1. The yield of oxidative multimer formation was low and its statistics were further determined by the evaluation of representative full-sized images (Figure S3 and Tables S3–S6) as exemplified for the MSP1D1_N42C variant in Figure 4. Dimer and trimer formation was evidenced by atomic force microscopy (AFM) for both MSP1D1_N42C and the double mutant (Figure 5A,B) with a height scale of 5 nm. The structures could be distinguished from accidental neighbors by their common displacement under the AFM tip upon multiple scans of identical sample areas. This is exemplified by the time-lapse video S1 in the Supplementary Material, showing a stable dimer over 15 min. The images comply with the expected lateral linkage of the rims of adjacent nanodiscs with the bilayer planes oriented parallel to the mica substrate and a bilayer thickness of ~4 nm in agreement with structural models [25,26] and AFM measurements in related systems [36]. This arrangement is represented in the molecular model in Figure 5C. No indication for transversally stacked multimers was found either in the top or side view. Steric hindrance between adjacent nanodiscs is probably the main reason for the low yield. However, the trimers observed here provide a reference for the most closely spaced covalent lateral packing that can be achieved.

To allow more freedom in the design of multimeric structures with longer and variable linkers, we used oligonucleotides connected to nanodisc cysteines through a maleimide–thiol reaction. Two complementary oligos carrying a maleimide group at the 5′ end (Table S2) were reacted with the pure MSP1D1 variants under reducing conditions (10 mM of TCEP). Nanodiscs were then produced from these two preparations and mixed in a 1:1 ratio which led to the formation of nanodisc multimers as a consequence of DNA hybridization. The pyrene experiments showed the thiol accessibility for all MSP1D1 variants but did not imply specific geometrical features upon reaction with the maleimide-labelled oligos. Furthermore, the flexibility of the adduct was purposefully increased by a 5′ linker of four thymines preceding 30 bases for nanodisc hybridization, which correspond to three helical turns of dsDNA and can be regarded as a stiff rod [37]. Despite the unpredictable geometry of the final dsDNA helix with respect to the nanodiscs, the homodimeric protein scaffold predicts clear relations between the orientation of the two DNA helices attached to either cysteine in single cysteine mutants of MSP1D1. Figure 1C shows the MSP1D1 structure with the cysteine mutation introduced at position 42 in its standard rotamer state. The structural freedom of any cysteine-linked modification in MSP1D1_N42C would in the most general case (azimuthal and polar angles different from zero) define a cone of steric angles on both sides of the nanodisc with antiparallel orientation of their cone axes (e.g., the dsDNA helical axes) relative to the bilayer normal. The actual arrangement of dsDNA-linked nanodiscs was investigated by transmission electron microscopy (TEM). Figure 5D shows TEM images of uranyl-formate-stained images of samples produced by hybridization of nanodiscs from two complementary oligonucleotide-modified MSP1D1_N42C preparations reconstituted with DMPG. The electron density suggests a side view of transversally stacked nanodiscs. Typically, the multimers showed a tendency to branch and run in roughly parallel bundles. Surprisingly, we did not observe gaps between individual nanodiscs that corresponded to the length of the linking 30 bp dsDNA (~10 nm). Instead, the nanodisc multimers appeared densely packed with very little space between them and with a contrast periodicity of ~4 nm along the stacking axis, indicative of the linking dsDNA being sandwiched between nanodiscs, as proposed in the structural model of the assembly shown in Figure 5E.

## 3. Discussion

Serial single molecule experiments as well as ensemble experiments on membrane proteins can profit from an ordered molecular assembly of these proteins. Membrane proteins can be exposed to identical experimental conditions in a super-molecular structure for serial interrogation by optical methods or their coherent scattering enforced for structural analyses by diffraction methods tailored to nano-sized objects [38,39]. Recent strategies in this direction have exploited the potential of DNA-hybridization-mediated assembling of nano-sized lipid bilayers. For example, the linkage of oligonucleotides to engineered cysteines in MSP-stabilized nanodiscs has been used for tethering nanodiscs to DNA-origamis [40]. Alternatively, crystallization of two longer apolipoproteins has been achieved [22]. Both approaches may ultimately circumvent the necessity of direct membrane protein crystallization for their structure determination. Finally, the self-assembly of MSP-based nanodiscs into one-dimensional stacks has been achieved for the design of nanomaterials [41,42]. In this case, the conceptual absence of membrane proteins allowed the enforcement of the transversal surface interaction between nanodiscs directly by the incorporation of DNA-modified lipids that were statistically partitioned in the bilayer.

Here, we have demonstrated an alternative way to induce dsDNA-mediated stacking of nanodiscs that exploits chemical modification of the MSP. Thereby, interference with lipid protein interactions can be prevented in applications that require native membrane protein structures within stacked nanodiscs as, for example, in single particle X-ray diffraction. Instead of lipid modification, the presented protocol generates long one-dimensional nanodisc multimers through hybridization between ssDNA attached to engineered cysteines in MSP1D1. The nanodisc monomers were indistinguishable from nanodiscs obtained with cysteine-free MSP1D1 with respect to DLS, CD and SEC analyses. The resulting stacks comprised more than hundred individual MSP-DNA units linked by three double-helical turns on either side of the nanodiscs. In contrast, only low yields of dimers and trimers were achieved when direct disulphide bond formation of three tested MSP1D1 variants was induced. The lateral alignment of disulphide-linked nanodiscs on a mica surface was obvious from AFM measurements showing the well-resolved height and outer circumference of the individual nanodiscs which agreed with those of free nanodiscs. This geometry was expected for sterical reasons, because the cysteine side chains are too short to bridge two nanodiscs in a transversally stacked geometry (Figure 1C). The AFM images may nevertheless not reflect the solution state, as we assume that the Mg^2+^ -mediated adsorption of the negatively charged lipids to mica stabilizes the “face-up” adsorption over a “side-up” geometry. In solution, rotational freedom is expected to allow large variability of the angle between the bilayer planes of linked nanodiscs.

The AFM images of dimers and trimers have been acquired primarily to provide a reference for the interpretation of the much larger DNA-linked multimeric assemblies seen in transmission electron microscopy (TEM). Here, the repeating units do not comply with the lateral alignment of circular bilayers but rather with the projection of transversally stacked bilayers that have their plane oriented perpendicular to the substrate surface. Consequently, the projection contrast shows stripes rather than lined-up circular discs. Although lower in resolution than the TEM images, AFM images also confirmed the formation of elongated structures in the same length range, as shown in Figure S5. The structure remained visible over a period of 38 min during which it was displaced under the influence of the AFM tip, as can be seen in the time-lapse AFM sequence (Video S2). Thus, stack formation was preserved under both experimental conditions. The presence of long multimeric structures under both imaging conditions is noteworthy, as it confirms the DNA-dependent stacking rather than uranyl stain-induced nanodisc interactions in TEM. In principle, negative stains can promote stacking of lipidic systems as shown for dimyristoyl-phosphatidyl-choline vesicles, for which uranyl formate was the preferred stain to prevent such “rouleau” artifacts [43].

The formation of stacks through dsDNA linkers was not strictly predictable. However, the most likely steric arrangement of DNA extensions was expected to exhibit opposite out-of-plane components on both sides of a nanodisc due to the antiparallel arrangement of MSP-monomers around the lipid bilayer (Figure 1C). Among the three tested MSPs, the MSP1D1-N42C variant was found to be the more suitable system for the one-dimensional stacking of nanodiscs in terms of expression yield and efficient cysteine modification. Remarkably, the observed stacks did not exhibit preferred bending. The cysteine of this variant was chosen to generate in the final nanodisc a pair of cysteines with large separation across the bilayer plain and little or no interference with lipid interactions. The predicted angle between the two cysteines is ~130° with respect to the center of the nanodisc. At 180°, a stacked monomer, i.e., one nanodisc with two oligonucleotides, would exhibit inversion symmetry. This would render the two half sides of each nanodisc in the multimeric assembly equivalent with respect to any dissections through the center of the lipid bilayer, thereby, providing little sterical preference for sidedness of bending. In combination with the out-of-plane orientation of the DNA helical axis, the large angle between the cysteines may thus explain why the TEM images revealed straight multimers of stacked nanodiscs, rather than arc-shaped or statistically curved aggregates expected for more attachment of the two DNA-linkers in close proximity. The observed stretches typically reached over 150 nanometers and were much longer and more uniform in thickness than those reported for nanodisc stacks formed by DNA-modified lipids [41,42]. Parallel runs of two stacked multimers were frequently observed (Figure 5D) and could be the consequence of mechanically-induced kinks or occasional deviations from the preferential antiparallel DNA orientation during the assembly process such that the addition of monomers at the free ends of the stacks becomes reversed sporadically. The latter notion is strongly supported by the existence of a small population of Cys-42 side chains that are oriented differently from the majority, as revealed by pyrene fluorescence (Figure 3). However, we have not found indications of the transition between lateral and transversal alignments. Thus, an orientation of the DNA helical axes pointing outward from the nanodisc and in plane with the bilayer appears to be rare or absent at all.

The proposed multimer model in Figure 5E accounts for the tight packing of monomers lacking a possible DNA-bridged gap of three helical turns (~10 nm). Consequently, the dsDNA is suggested to zigzag between monomers, with the helical axis virtually parallel to the bilayer plane, rather than acting as a spacer between nanodiscs. This brings the negatively charged DNA backbone close to the negatively charged DMPG bilayer surface which is counterintuitive. However, all experiments were performed in the presence of Mg^2+^ (10 mM) which is known to penetrate into the headgroup region of DMPG, thereby dehydrating it and neutralizing its phosphate charge [44]. Thus, the proposed DNA arrangement is actually fully compatible with the physical chemistry of the lipid–cation interactions in this system.

The DNA-mediated formation of nanodisc multimers can be extended to larger MSP variants and to DNA-encircled lipid bilayers (DEBs), where the lipid bilayer is encircled by DNA in the first place. An analogous supramolecular assembly strategy for polymer-bounded lipid bilayers would be attractive, as it could be combined with the detergent-free solubilization of membrane proteins [2,45], although the flexibility and chemical heterogeneity of the current systems [46,47] impose additional challenges. The method described here will allow one to vary the stack width of nanodisc multimers by tuning the dsDNA length to the spatial requirements of extra-membranous domains of membrane proteins. This adaptability renders the system attractive for crystallization-independent single particle-based diffraction studies of membrane proteins.

## 4. Materials and Methods

### 4.1. Molecular Cloning of MSP1D1 Cysteine Mutants

The gene encoding for MSP1D1 was synthesized by GeneArt (Regensburg, Germany) with optimized codon usage for the expression in *Escherichia coli* and cloned into the standard vector pET28a(+) from Novagen. The mutation of selected amino acids to cysteines (Figure 1A) was accomplished with the QuikChange Lightning Multi Site-Directed Mutagenesis Kit from Agilent according to the manual. The DNA sequence was verified by sequencing. The mutagenesis primers (Table S1) were designed with the online tool provided by the manufacturer (http://www.genomics.agilent.com/primerDesignProgram.jsp, accessed on 13 February 2021). The DNA sequence was verified by sequencing (GATC-Biotech/LIGHTrun) with the standard T7-primer and transformed into BL21 (DE3) gold expression cells according to manufacturer’s protocol (Novagen).

### 4.2. Expression and Purification of MSP1D1 Constructs

The standard expression of MSP1D1 [20] was adapted to the protocol from Inagaki et al. [48]. Briefly, BL21Gold (DE3) cells (Agilent Technologies, Santa Clara, CA, USA) carrying the original MSP1D1-pET28a plasmid, or one of three different cysteine-encoding MSP1D1-pET28a plasmids, were grown in at 37 °C in defined 2xYT medium Broth containing 50 μg/mL of kanamycin overnight. After induction with 0.3 mM of isopropyl-β-d-thiogalactopyranoside (IPTG), the temperature was decreased to 28 °C. The cells were harvested 4 h later, frozen in liquid nitrogen, and stored at −80 °C until further use. All the following steps were carried out at 4 °C. For purification, cells were re-suspended in buffer A (50 mM of Tris-HCl, 200 mM of NaCl, pH 7.4), protease inhibitors (Roche Applied Science, Penzberg, Germany), 1% Triton X-100 and 12 μg/mL of DNase. After mechanical disruption, the cell homogenate was centrifuged at 15,000× *g* for 70 min to remove cell debris. Imidazole was added to the supernatant to a final concentration of 25 mM. The sample was loaded onto a Ni-NTA column (GE-Healthcare, Chicago, IL, USA), equilibrated with buffer A containing 25 mM of imidazole). The column was washed with different buffer solutions: (a) buffer A with 1% Triton X-100, (b) buffer A with 25 mM of sodium cholate, (c) buffer A with 10 mM of ATP/MgCl_2_ and (d) buffer A with 25 mM of imidazole. Finally, the protein was eluted by gradient elution using increasing concentrations of imidazole (280 mM, 500 mM and 1 M). Fractions containing MSP1D1 were identified by 15% SDS-PAGE. Imidazole was removed by a desalting step (PD10 column, equilibrated with buffer A; GE Healthcare) and concentrated using Vivaspin4 columns (Satorius, Göttingen, Germany). The final protein concentration was determined by measuring the absorbance at 280 nm with a NanoDrop spectrophotometer using a calculated extinction coefficient of 21,430 M^−1^ cm^−1^ and a calculated molecular mass of 24,793 Da (ProtParam, ExPASy). All MSP1D1 variants were tested on a 15% SDS-PAGE under reducing (1 mM of tris(2-carboxyethyl)phosphine (TCEP) and non-reducing (without TCEP)) conditions (Figure S1). The purified MSP1D1 variants were frozen in liquid nitrogen and stored at −80 °C until further use.

### 4.3. Assembly of Lipid Nanodiscs

Nanodiscs of the different MSP1D1 constructs were prepared with 1, 2-dimyristoyl-*sn*-glycero-3-phospho-(1′-rac-glycerol) (DMPG) (Avanti Polar Lipids) according to the original protocol [19]. For the assembly, a vacuum-dried DMPG lipid film was solubilized in Buffer A, containing cholate at twice the lipid concentration and sonicated until a clear solution was obtained. The lipid/sodium cholate solution and the MSP1D1 variants were mixed to give final concentrations of 12 mM and 0.2 mM, respectively. The mixture was incubated for 1 h at 25 °C. The detergent was removed by detergent removal spin columns (Pierce). The nanodisc solution was centrifuged at 11,000× *g* (10 min) to remove aggregates. The size and the homogeneity of the different MSP1D1 nanodiscs were verified by size exclusion chromatography (SEC) with a Superdex 200 Increase 10/300 GL column (GE Heathcare). The fractions corresponding to the retention volume of nanodiscs were checked for purity using SDS-PAGE (Figure S1).

### 4.4. Dynamic Light Scattering and Circular Dichroism

Dynamic light scattering (DLS) was measured with a Zetasizer Nano (Malvern Instruments Ltd., Malvern, UK) at room temperature. The translational diffusion coefficients and corresponding hydrodynamic radii D_H_ were derived from an autocorrelation analysis of the quasi-elastically back-scattered 632.8 nm light measured at an angle of 173°. Nanodisc samples (7.5 µM in buffer A) were centrifuged at 10,000× *g* for 10 min before measurements. Circular dichroism (CD) spectra of purified nanodiscs (5 µM in buffer A) were measured at room temperature in a 1 mm cuvette with a J-815 spectrometer (JASCO) between 185 and 320 nm at a scan rate of 100 nm/min (band width 4 nm). Spectra were averaged over eight individual measurements.

### 4.5. Pyrene Maleimide Fluorescent Labelling of Cysteines

Nanodisc samples under reducing conditions (1 mM of TCEP) were treated with 10-fold molar excess of N-(1-pyrene) maleimide (TCI Deutschland GmbH, Eschborn, Germany) for 16 h at 4 °C as recommended [30]. The labelled MSP1D1 variants were purified by size exclusion chromatography (SEC) and used for nanodisc assembly. Fluorescence emission spectra (excitation at 345 nm) were recorded on an LS 55 luminescence spectrofluorometer (Perkin Elmer, Waltham, MA, USA) in 1 cm × 1 cm reduced volume Suprasil quartz glass cuvettes (Hellma, Müllheim, Germany) at a scan rate of 120 nm/minute and 5 nm bandwidth.

### 4.6. Maleimide-Modified Oligonucleotides for Nanodisc Linkage

Oligonucleotides (oligos) comprising 34 bases and a 5′- maleimide modification, separated by a two-carbon spacer, were purchased from Genelink (Table S2). The protected maleimide group was activated according to the provided protocol from Genelink (http://www.genelink.com/newsite/products/mod_detail.asp?modid=226, accessed on 13 February 2021). Nanodiscs (5 µM) formed by the MSP1D1_N42C variant were incubated with the maleimide-modified oligos in buffer A in the presence of 10 mM of TCEP at 20 °C for 1 h, to reduce the thiols of cysteines for the interaction. Unreacted oligos were removed by Ni-NTA purification. Two populations—ND_O1 and ND_O2—of nanodiscs were formed with oligo1- and oligo2-modified MSP1D1_N42C, respectively. Aliquots of ND_O1 and ND_O2 were mixed at a ratio of 1:1 and incubated at 20 °C to allow dsDNA formation between nanodiscs.

### 4.7. Atomic Force Microscopy

Nanodiscs (2.5 µM) were diluted (1:40) in 10 mM of ammonium acetate buffer containing 10 mM of MgCl_2_. 50µL of the sample solution were pipetted on freshly cleaved mica substrate. Imaging by atomic force microscopy (AFM) was performed using a Cypher ES (Asylum Research, Goleta, CA, USA) operated in tapping mode with a Biolever Mini BL-AC40TS-C2 cantilever (Olympus, Tokyo, Japan). Images were recorded with a scan size of 2 × 2 µm^2^, at a line rate of 1 Hz and a resolution of 1024 × 1024 px^2^ and AFM video S2 was recorded at the same pixel resolution but with a scan size of 0.5 × 0.5 µm^2^ and a line rate of 4 Hz. Video S1 was recorded with a scan size of 150 × 150 nm at 256 × 256 px^2^ resolution and with a line rate of 10 Hz. Data processing was carried out in four consecutive steps using the software Gwyddion 2.55: 1. Data levelling (plane levelling): level data by mean plane subtraction, 2. Scan line artifacts (remove scars): correct horizontal scars (strokes), 3. Align rows (align rows using various methods) median, 4. Polynomial background (remove polynomial background): horizontal and vertical polynomial degrees of 2.

### 4.8. Transmission Electron Microscopy

Samples were additionally studied by transmission electron microscopy (TEM) using the undiluted nanodisc preparation for incubation (1 min) on a 300-mesh carbon grid. For negative staining, 2% uranyl formate was used, and TEM images obtained with a Morgagni 268 microscope operated at 80 kV.

### 4.9. Structure Modelling

The structures of DMPG-filled MSP1D1 nanodiscs (Figure 1B–E) was generated with CHARMM-GUI Nanodisc Builder [25]. Cysteine replacements were introduced using Pymol [27]. For modelling of the DNA-linkage to the thiols in cysteine-carrying nanodiscs, the structure of the corresponding dsDNA was placed such that the reactive carbon atom of the 5′-maleimide-modification was in bond length distance to the thiol sulfur in the cysteines. Short Molecular Dynamics (MD) simulations (5 ns) were performed on the oligo using the AMBER15 [49] program package with the ff99bsc0+OL15 force field applied to the oligo, after which the maleimide modification was added manually.

## Data Availability

Not available.

## Supplementary Materials

Figure S1: SDS-Polyacrylamide gel electrophoresis of MSP1D1 variants, Figure S2: Size exclusion chromatography of nanodiscs formed by MSP1D1 cysteine variants, Figure S3: AFM analysis of copper-induced oligomerization of nanodiscs, Figure S4: Yield analysis of copper-catalyzed intermolecular disulphide bond formation between MSP1D1_N42C- and MSP1D1_N42C/K163C-derived nanodiscs, Figure S5: Nanodisc stacks—Selected AFM images of a series recorded over a time of 38.5 min, Table S1: Overview of site-directed mutagenesis primers for MSP1D1 cysteine mutants, Table S2: Maleimide-modified oligos used for nanodisc linkage, Table S3: AFM image evaluation of nanodisc samples from MSP1D1_N42C + Cu, Table S4: AFM image evaluation of nanodisc samples from MSP1D1_N42C/K163C + Cu, Table S5: AFM image evaluation of nanodisc samples from MSP1D1_N42C (in TCEP), Table S6: Summary of statistical analysis of copper-induced oligomerization in MSP1D1 variants, Video S1: Time-lapse AFM sequence of nanodiscs moving under the AFM tip as linked dimers, Video S2: Time-lapse AFM sequence of the nanodisc stacks in Figure S5 observed over 38.5 min.

## Sample Availability

Not available.
