# Supplementary material for: DNA-Mediated Stack Formation of Nanodiscs
Source: Molecules. 2021 Mar 16;26(6):1647. doi: 10.3390/molecules26061647 (PMC8000961; doi:10.3390/molecules26061647)
Supplement: Supplementary file 1 [file molecules-26-01647-s001.zip › molecules-1128715-SM/molecules-1128715-SM.pdf]

DNA-mediated stack formation of nanodiscs

Madhumalar Subramanian [1,2], Charlotte Kielar [1], Satoru Tsushima [1], Karim Fahmy [1,2,*], and Jana Oertel [1,*]

[1] Helmholtz-Zentrum Dresden-Rossendorf, Institute of Resource Ecology, Biophysics Department, Bautzner Landstrasse 400, 01328 Dresden, Germany; m.subramanian@hzdr.de (M.S.); c.kielar@hzdr.de (C.K.); s.tsushima@hzdr.de (S.T); k.fahmy@hzdr.de (K.F); j.oertel@hzdr.de (J.O)

[2] Technische Universität Dresden, Center for Molecular and Cellular Bioengineering;

* Correspondence: k.fahmy@hzdr.de; j.oertel@hzdr.de; Tel.: +49-351-260-2952 (K.F.); +49-351-260-2978 (J.O.)

SUPPLEMENTARY MATERIALS

Figure S1: SDS-Polyacrylamide gel electrophoresis of MSP1D1 variants

Figure S2: Size exclusion chromatography of nanodiscs formed by MSP1D1 cysteine variants

Figure S3: AFM analysis of copper-induced oligomerization of nanodiscs

Figure S4: Yield analysis of copper-catalyzed intermolecular disulphide bond formation between MSP1D1_N42C and MSP1D1_N42C/K163C-derived nanodiscs

Figure S5: Nanodisc stacks - Selected AFM images of a series recorded over a time of 38.5 min

Table S1: Overview of site-directed mutagenesis primers for MSP1D1 cysteine mutants

Table S2: Maleimide-modified oligos used for nanodisc linkage

Table S3: AFM image evaluation of nanodisc samples from MSP1D1_N42C + Cu

Table S4: AFM image evaluation of nanodisc samples from MSP1D1_N42C/K163C + Cu

Table S5: AFM image evaluation of nanodisc samples from MSP1D1_N42C (in TCEP)

Table S6: Summary of statistical analysis of copper-induced oligomerization in MSP1D1 variants

Video S1: Time-laps AFM sequence of nanodiscs moving under the AFM tip as linked dimers

Video S2: Time-laps AFM sequence of the nanodisc stacks in Figure S5 observed over 38.5 min

Expression and purification of MSP1D1 variants

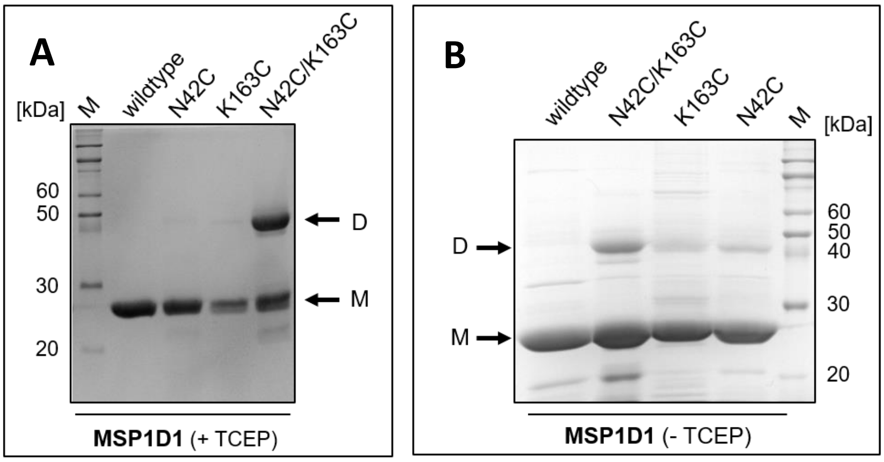

Figure S1. SDS-Polyacrylamide gel electrophoresis of MSP1D1 variants. (A) Under reducing conditions (1 mM TCEP),the two single cysteine variants migrated as monomers (25 kDa), whereas the double mutation MSP1D1_N42C/K163C showed an additional band at 50 kDa corresponding to dimers (D). (B) Under non-reducing conditions (-TCEP), all MSP1D1 variants migrated as monomers at 25 kDa (M) and as covalently linked dimers at 50 kDa (D). The cysteine-free MSP1D1 form showed only the monomer band. Gels (15%) were stained with Coomassie blue.

Nanodisc assembly of MSP1D1 cysteine variants

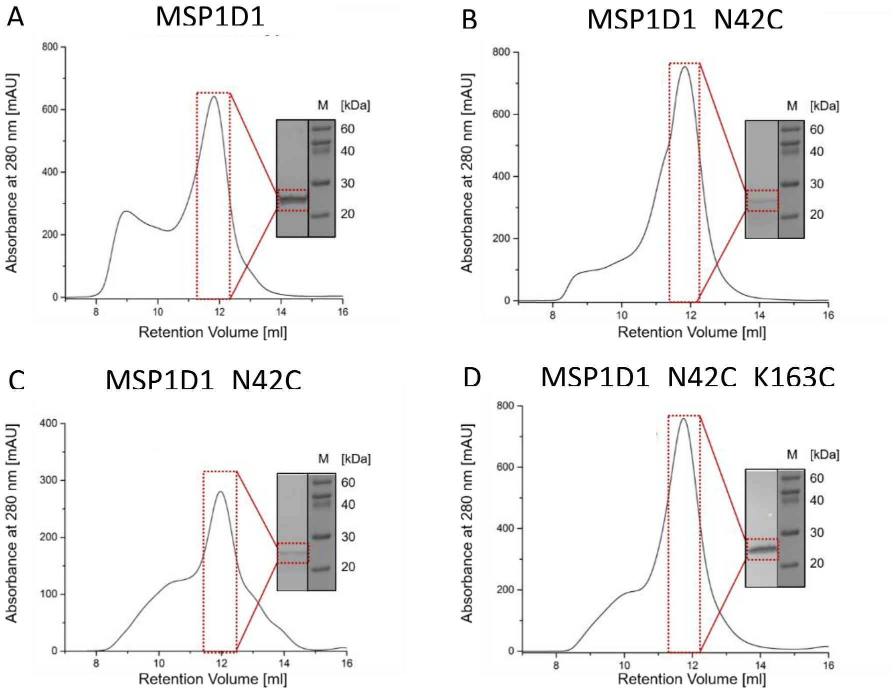

Figure S2. Size exclusion chromatography of nanodiscs formed by MSP1D1 cysteine variants. The elution of DMPG-filled nanodiscs was monitored by 280 nm absorption under reducing conditions in the presence of 1 mM TCEP as reducing agent. The indicated fractions at the retention volume of MSP1D1 (red rectangle) were collected and analyzed by SDS page, where they migrated with the expected molecular mass of 25 kDa. The lower 280 nm absorption and weaker gel stain for MSP1D1_K163C corresponded to the lower amount of loaded material for this variant which showed a lower expression yield.

AFM imaging of copper-induced oligomerization of MSP1D1_N42C and MSP1D1_K163C nanodiscs.

Copper-induced oxidative disulphide bond formation generated dimers and trimers with all three MSP1D1 variants (Figure S3, upper panel). The statistics of oligomer formation by the three MSP1D1 variants was based on manual counting over large areas of AFM images as exemplified (Figure S3, lower panel). MSP1D1_N42C nanodiscs exhibited oxidative dimer formation at a percentage of 9.0% (± 1.4) and trimer formation at 0.3% (± 0.2). These fractions made up 9.3% (± 0.8) and 0.3% (± 0.2), respectively, for nanodiscs formed with

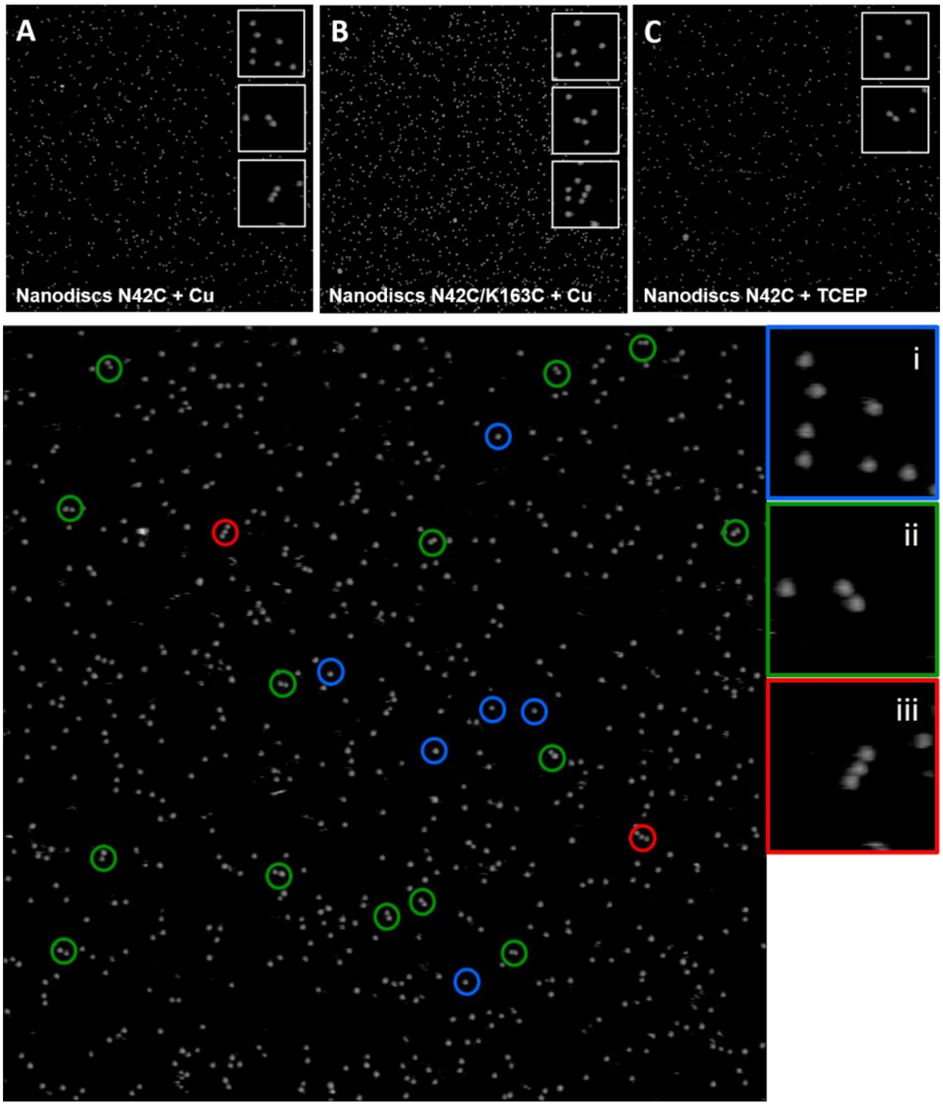

Figure S3. AFM analysis of copper-induced oligomerization of nanodiscs. Upper panel: MSP1D1_N42C (A) and MSP1D1_N42C/K163C nanodiscs (B) were incubated with Cu^{2+} at a ratio of 1:20 (MSP1D1 : Cu) to induce oligomerization of nanodiscs by copper-catalyzed disulphide bond formation between nanodiscs. (C) As a negative control, MSP1D1_N42C nanodiscs were also imaged under reducing conditions (10 mM TCEP). Lower panel: The example of manual nanodisc counting of liquid mode AFM images shown in Figure 4 in the main text is presented here again for comparison**.** Monomers, dimers and trimers of nanodiscs are encircled in blue, green and red, respectively, and shown enlarged on the right. Data are from the MSP1D1_N42C variant. All images displayed with a size of 2x2 μm^2 and a height scale of 7.5 nm.

MSP1D1_N42C/K163C. For comparison, the negative control (MSP1D1_N42C under reducing conditions, 1mM TCEP) showed a residual dimerization of 4.2% (± 1.29). Manual counting was carried out on individual images (Figure S4) and the results summarized in Table 4. The final multimer yields of the samples were determined by averaging over the individual yields of the AFM images with the corresponding standard deviation as a measure of the error (Tables S3-S6).

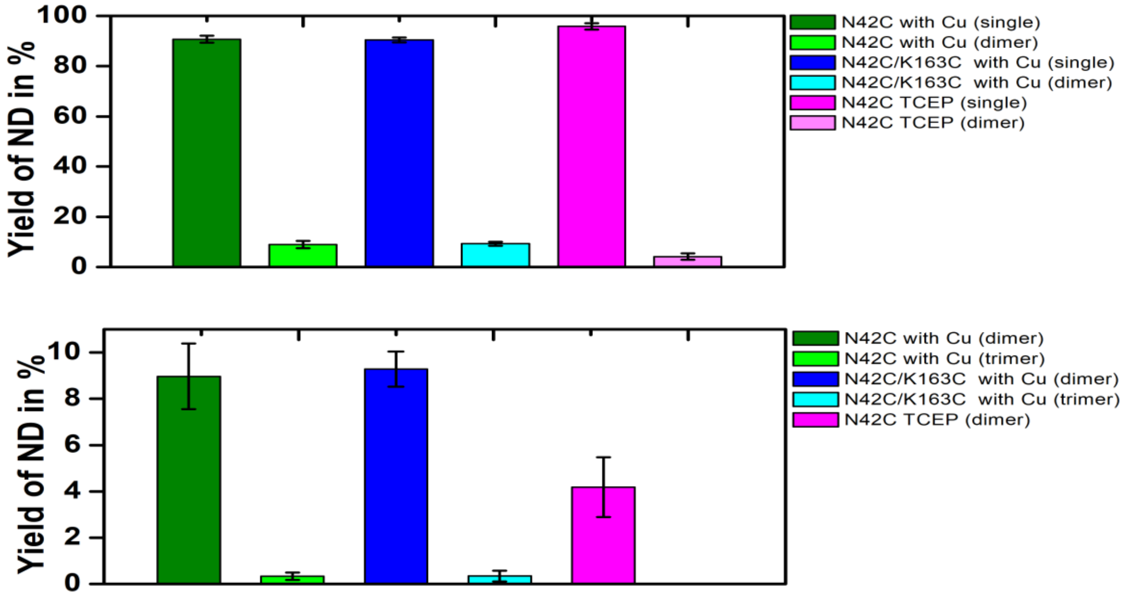

Figure S4. Yield analysis of copper-catalyzed intermolecular disulphide bond formation between MSP1D1_N42C- and MSP1D1_N42C/K163C-derived nanodiscs. Upper panel: single nanodiscs and nanodisc dimers. MSP1D1_N42C nanodiscs in TCEP without copper served as a negative control. Lower panel: nanodisc dimers and trimers.

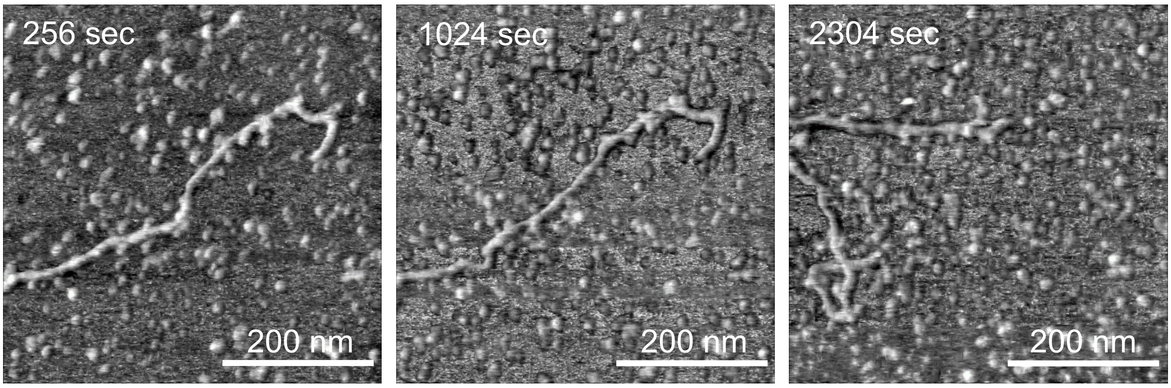

Figure S5. Nanodisc stacks - Selected AFM images of a series recorded over a time of 38.5 min. The images have been recorded in intermittent contact mode at a line rate of 4 Hz with a scan area of 0.5x0.5µm² at a resolution of 1024x1024 px². The recorded images indicate that the thread-like structure remains intact during scanning but is repositioned by tip sample interactions.

TABLES

Table S1. Overview of site-directed mutagenesis primers for MSP1D1 cysteine mutants.

substituted residues	Primer	template	Construct name
N42C	Primer N42C<br>5'-ccttcggtttctttttccagacaatcccaaaattcttgggtaaccg-3'	MSP1D1	MSP1_N42C
K163C	Primer K163C<br>5'-cccgtctggccgaatatcatgcatgcgcaaccgaacatctgag-3'	MSP1D1	MSP1_K163C
N42C/ K163C	Primer N42C + Primer K163C	MSP1D1	MSP1_N42C/K163C

Table S2. Maleimide-modified oligos used for nanodisc linkage.

**Oligo1-Mal**
5'-Mal-(2 carbon spacer)- TTTTACATTCTACTTACCTTCTCTATACTCTTCC -3'
**Oligo2-Mal**
5'-Mal-(2 carbon spacer)- TTTTGGAAGAGTATAGAGAAGGTAAGTAGAATGT -3’

Table S3. AFM image evaluation of nanodisc samples from MSP1D1_N42C + Cu^{2+}. Number of manually evaluated nanodiscs ($N_{(ND)}$) and the corresponding yields of single, dimeric and trimeric nanodiscs in %.

AFM image	$ND_{(single)}$	$ND_{(dimer)}$	$ND_{(trimer)}$	$N_{(ND)}$
1	89.55	10.28	0.17	574
2	92.20	7.46	0.34	590
3	93.33	9.17	0.50	600
**Sum**	**90.69 ± 1.36**	**8.97 ± 1.42**	**0.34 ± 0.16**	**1764**

Table S4. AFM image evaluation of nanodisc samples from MSP1D1_N42C/K163C + Cu^{2+}. Number of manually evaluated nanodiscs (N(ND)) and the corresponding yields of single, dimeric and trimeric nanodiscs in %.

AFM image	$ND_{(single)}$	$ND_{(dimer)}$	$ND_{(trimer)}$	$N_{(ND)}$
1	89.28	10.16	0.56	1063
2	91.01	8.90	0.09	1079
3	90.84	8.79	0.37	1070
**Sum**	**90.38 ± 0.96**	**9.28 ± 0.76**	**0.34 ± 0.24**	**3212**

Table S5. AFM image evaluation of nanodisc samples from MSP1D1_N42C (in TCEP). Number of manually evaluated nanodiscs (N(ND)) and the corresponding yields of single, dimeric and trimeric nanodiscs in %.

AFM image	$ND_{(single)}$	$ND_{(dimer)}$	$N_{(ND)}$
1	94.89	5.11	587
2	97.29	2.71	517
3	95.26	4.74	570
**Sum**	**95.81 ± 1.29**	**4.19 ± 1.29**	**1674**

Table S6. Summary of statistical analysis of copper-induced oligomerization in MSP1D1 variants.

	single ND in %	dimer ND in %	trimer ND in %
MSP1D1_N42C + Cu^{2+}	90.69 ± 1.36	8.97 ± 1.42	0.34 ± 0.16
MSP1D1_N42C/K163C + Cu^{2+}	90.38 ± 0.96	9.28 ± 0.76	0.34 ± 0.24
MSP1D1_N42C + TCEP	95.81 ± 1.29	4.19 ± 1.29	

Video S1: Time-laps AFM sequence of nanodiscs moving under the AFM tip as linked dimers. The images have been recorded in intermittent contact mode over 15 min in a scan area of 150 nm x 150 nm at a line rate of 10 Hz and resolution of 256 x 256 px^2.

Video S2: Time-laps AFM sequence of the nanodisc stacks in Figure S5 observed over 38.5 min. The images have been recorded in intermittent contact mode at a line rate of 4 Hz with a scan area of 0.5x0.5µm² at a resolution of 1024x1024 px².
